# Integrated Multi-Omics Analyses in Oncology: A Review of Machine Learning Methods and Tools

**DOI:** 10.3389/fonc.2020.01030

**Published:** 2020-06-30

**Authors:** Giovanna Nicora, Francesca Vitali, Arianna Dagliati, Nophar Geifman, Riccardo Bellazzi

**Affiliations:** ^1^Department of Electrical, Computer and Biomedical Engineering, University of Pavia, Pavia, Italy; ^2^Center for Innovation in Brain Science, University of Arizona, Tucson, AZ, United States; ^3^Department of Neurology, College of Medicine, University of Arizona, Tucson, AZ, United States; ^4^Center for Biomedical Informatics and Biostatistics, University of Arizona, Tucson, AZ, United States; ^5^Centre for Health Informatics, The University of Manchester, Manchester, United Kingdom; ^6^The Manchester Molecular Pathology Innovation Centre, The University of Manchester, Manchester, United Kingdom

**Keywords:** multi-omics, machine learning, tools, systematic review, oncology, cancer

## Abstract

In recent years, high-throughput sequencing technologies provide unprecedented opportunity to depict cancer samples at multiple molecular levels. The integration and analysis of these multi-omics datasets is a crucial and critical step to gain actionable knowledge in a precision medicine framework. This paper explores recent data-driven methodologies that have been developed and applied to respond major challenges of stratified medicine in oncology, including patients' phenotyping, biomarker discovery, and drug repurposing. We systematically retrieved peer-reviewed journals published from 2014 to 2019, select and thoroughly describe the tools presenting the most promising innovations regarding the integration of heterogeneous data, the machine learning methodologies that successfully tackled the complexity of multi-omics data, and the frameworks to deliver actionable results for clinical practice. The review is organized according to the applied methods: Deep learning, Network-based methods, Clustering, Features Extraction, and Transformation, Factorization. We provide an overview of the tools available in each methodological group and underline the relationship among the different categories. Our analysis revealed how multi-omics datasets could be exploited to drive precision oncology, but also current limitations in the development of multi-omics data integration.

## Introduction

The integration and analysis of high-throughput molecular assays is a major focus for precision medicine in enabling the understanding of patient and disease specific variations. Integrated approaches allow for comprehensive views of genetic, biochemical, metabolic, proteomic, and epigenetic processes underlying a disease that, otherwise, could not be fully investigated by single-omics approaches. Computational multi-omics approaches are based on machine learning techniques and typically aim at classifying patients into cancer subtypes (1–5), designed for biomarker discovery and drug repurposing (6, 7).

While complexities underling cancer still hampers our understanding of how this disease arises and progresses (8), multi-omics approaches have been suggested as promising tools to dissect patient's dysfunctions in multiple biological systems that may be altered by cancer mechanisms (9).

Several efforts have been made to generate comprehensive multi-omics profiles of cancer patients. The Cancer Genome Atlas (TCGA, https://portal.gdc.cancer.gov/) provides detailed clinical, genomics, transcriptomics, and proteomics data on about 20,000 subjects and plans to generate additional data in the next years for a variety of cancer types. Analysis of datasets generated by multi-omics sequencing requires the development of computational approaches spanning from data integration (10), statistical methods, and artificial intelligence systems to gain actionable knowledge from data.

Here we present a descriptive overview on recent multi-omics approaches in oncology, which summarizes current state-of-art in multi-omics data analysis, relevant topics in terms of machine learning approaches, and aims of each survey, such as disease subtyping, or patient similarity. We provide an overview on each methodology group, while then focusing on publicly available tools.

## Methods

### Search Strategy

We retrieved publications by querying the Scopus database as: (cancer OR tumor OR tumor OR oncolog^*^)AND(multi-omic^*^ OR multiomic^*^OR mixomic^*^)AND(“machine learning” OR “data fusion” OR “network analysis”).

### Eligibility Criteria

Since other review covered previous years (10, 11) we included peer-reviewed journal articles published from 2014 to 2020 (last query 04-22-2020). If a study appears in multiple publications, only the latest version was included. We selected relevant studies by screening titles and abstracts, then analyzing full-texts. We excluded papers accordingly to the following criteria:

- Review articles;
- Studies focused on non-human subjects;
- Studies intended to validate and/or apply previously developed tools;
- Studies published in conference proceedings.
- Studies that integrate different measurement of the same type of omics (such as, only proteomics measurement).

### Categories and Analyses

For each article, we extracted the publication year and the number of citations. We categorize the selected publications according to:

- Data inputs (i.e., types of omics);
- Research Aims:
  1. Stratified Medicine for subgroup discovery: studies aimed at finding groups of patients that exhibit different therapeutic/prognostic outcomes;
  2. Biomarker discovery: studies that detect -omics characteristics indicating a disease state;
  3. Pathways analysis: studies aimed at discovering relation among -omics terms, such as genes or proteins in normal and cancer condition;
  4. Drug repurposing/discovery: studies aimed at identifying new drugs to or existing effective drugs originally developed for other conditions;
- Methods and algorithms: Deep network, Networks-based methods (Bayesian and Heuristic Networks), Clustering, Features Extraction, Feature Transformation, Factorization.

We highlight successful approaches for each criterion and identify promising ones that are either nascent or unexplored as potential opportunities.

## Results

**Figure 1:**
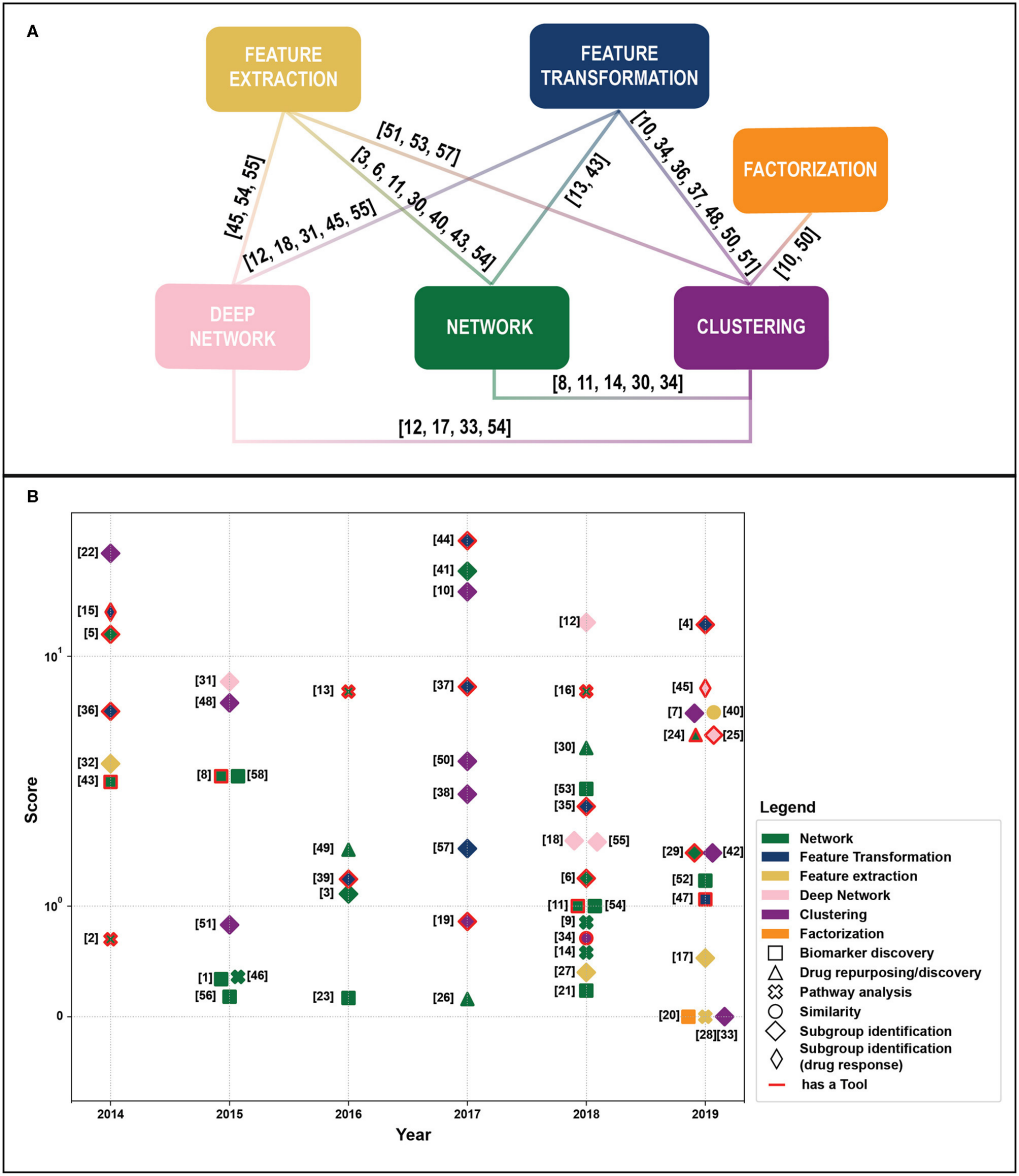
**(A)** Linkage between different methodological categories. References to papers (see Table 1). That could be categorized in different groups are reported near the link. **(B)** Publications by year of publication and Field-Weighted Citation Impact. Different colors indicate exploited methods, shapes aims, and outputs. Papers with red borders have source code or provide a tool. Papers in the “Subgroup identification” group and/or with free tool result to be the most cited across years. The reference numbers are reported in Table 1.

We retrieved 270 papers. The Scopus query did not retrieve 24 relevant works that were added manually based on our previous knowledge. After a screening of papers' abstracts, 58 papers meeting our criteria were selected. Retrieved papers were organized into a matrix table (Table 1) and analyzed with respect to the aforementioned categories. As highlighted in Figure 1A, categories are not mutually exclusive, thus we show links between groups, which relate papers applying multiple methods. Figure 1B depicts all considered publications by year of publication and the Field-Weighted Citation Impact, a metric that allows comparison of papers accounting for year of publication and number citations. Studies are shown with different colors and shapes according to method used and the aim/output type.

**Table 1:** Selected papers and categories.

**References**	**References in Figure 1**	**Year**	**#Citation 22/04/2020**	**Scopus field-weighted citation impact**	**Method**	**Omics**	**Aim**	**Tool release**
Agarwal et al. (12)	1	2015	2	0.34	Network	Genomics, transcriptomics	Biomarker discovery	
Amar and Shamir (13)	2	2014	16	0.70	Network	Proteomics, genomics	Pathways analysis	ModMap tool
Ao et al. (14)	3	2016	17	1.11	Network	Genomics, epigenomics	Subgroup identification	
Argelaguet et al. (15)	4	2019	57	14.40	Feature transformation	Transcriptomics, genomics	Subgroup identification	R package *MOFAtools*
Wang et al. (16)	5	2014	410	12.89	Network	Transcriptomics, epigenomics	Subgroup identification	R and MATLAB code http://compbio.cs.toronto.edu/SNF/
Beal et al. (17)	6	2018	2	1.25	Network	Transcriptomics, genomics	Subgroup identification	https://github.com/sysbio-curie/PROFILE
Benfeitas et al. (18)	7	2019	9	5.17	Clustering	Transcriptomics, proteomics, metabolomics	Subgroup identification	
Bonnet et al. (19)	8	2015	29	2.50	Network	Genomics, transcriptomics	Biomarker discovery	Lemon-Tree—command-line tool in Java http://lemon-tree.googlecode.com
Cancemi et al. (20)	9	2018	4	0.82	Network	Transcriptomics, proteomics	Pathways analysis	
Cavalli et al. (21)	10	2017	213	21.09	Clustering	Epigenomics, genomics, transcriptomics	Subgroup identification	
Champion et al. (22)	11	2018	6	1	Network	Genomics, epigenomics	Biomarker discovery	AMARETTO R package https://bitbucket.org/gevaertlab/pancanceramaretto
Chaudhary et al. (23)	12	2018	82	14.79	Deep network	Transcriptomics, epigenomics	Subgroup identification	
Cho et al. (24)	13	2016	48	6.65	Network	Genomics, proteomics	Pathways analysis	Mashup tool MATLAB code http://cb.csail.mit.edu/cb/mashup/
Costa et al. (25)	14	2018	4	0.58	Network	Genomics, epigenomics	Pathways analysis	
Costello et al. (26)	15	2014	271	14.12	Feature transformation	Genomics, transcriptomics, epigenomics, proteomics	Subgroup identification (drug response)	
Dimitrakopoulos et al. (27)	16	2018	29	6.67	Network	Genomics, transcriptomics, proteomics	Pathway analysis	https://github.com/cbg-ethz/netics
Drabovich et al. (28)	17	2019	1	0.53	Feature extraction	Transcriptomics, proteomics, secretomics, tissue specific	Subgroup identification	
Francescatto et al. (29)	18	2018	6	1.59	Deep network	Genomics, transcriptomics	Subgroup identification	
Gabasova et al. (30)	19	2017	6	0.86	Clustering	Transcriptomics, proteomics, epigenomics	Subgroup identification	Clusternomics R package https://github.com/evelinag/clusternomics
Gao et al. (31)	20	2019	0	0	Factorization	Transcriptomics, genomics	Biomarker discovery	
Griffin et al. (32)	21	2018	1	0.29	Network	Transcriptomics, epigenomics	Biomarker discovery	
Hoadley et al. (33)	22	2014	668	32.88	Clustering	Proteomics, transcriptomics, genomics	Subgroup identification	
Hua et al. (34)	23	2016	2	0.17	Network	Genomics, epigenomics	Biomarker discovery	
Huang et al. (35)	24	2019	6	4.44	Network	Genomics, transcriptomics, epigenomics	Drug repurposing/discovery	DrugComboExplorer tool https://github.com/Roosevelt-PKU/drugcombinationprediction
Huang et al. (36)	25	2019	8	4.37	Deep network	Transcriptomics	Subgroup identification	SALMON source code https://github.com/huangzhii/SALMON/
Kim et al. (37)	26	2017	3	0.16	Network	Transcriptomics, proteomics	Drug repurposing/discovery	
Kim et al. (38)	27	2018	2	0.40	Feature extraction	Genomics, transcriptomics, epigenomics	Subgroup identification	
Kim et al. (39)	28	2019	0	0	Feature extraction	Genomics, transcriptomics	Pathways analysis	
Koh et al. (40)	29	2019	2	1.48	Network	Transcriptomics, proteomics	Subgroup identification	iOmicsPASS https://github.com/cssblab/iOmicsPASS
Lee et al. (41)	30	2018	21	3.46	Network	Genomics, transcriptomics	Drug repurposing/discovery	
Liang et al. (42)	31	2015	86	5.96	Deep network	Transcriptomics, epigenomics	Subgroup identification	
List et al. (3)	32	2014	20	2.51	Feature extraction	Transcriptomics, epigenomics	Subgroup identification	
Luo et al. (43)	33	2019	0	0	Clustering	Transcriptomics, genomics	Subgroup identification	
Ma and Zhang (44)	34	2018	4	0.71	Clustering	Transcriptomics, epigenomics	Similarity	AFN is part of the Bioconductor R package https://bioconductor.org/packages/release/bioc/html/ANF.html
Mariette and Villa-Vialaneix (45)	35	2018	8	1.90	Feature transformation	Transcriptomics, genomics	Subgroup identification	R package *mixKernel*
Meng et al. (46)	36	2014	79	5.29	Feature transformation	Transcriptomics, proteomics	Subgroup identification	R package *omicade4*
Mo et al. (47)	37	2017	18	7.03	Feature transformation	Transcriptomics, genomics	Subgroup identification	R package *iClusterPlus*
Nguyen et al. (48)	38	2017	20	2.03	Clustering	Transcriptomics, epigenomics, genomics	Subgroup identification	
O'Connell and Lock (49)	39	2016	13	1.21	Feature transformation	Transcriptomics, genomics	Subgroup identification	R Package *r.jive*
Pai et al. (50)	40	2019	6	5.23	Feature extraction	Transcriptomics, metabolomics, genomics	Similarity	
Raphael et al. (51)	41	2017	269	26.77	Network	Transcriptomics, genomics, proteomics	Subgroup identification	
Rappoport et al. (52)	42	2019	2	1.48	Clustering	Transcriptomics, epigenomics	Subgroup identification	
Ray et al. (4)	43	2014	30	2.34	Bayesian network	Genomics, epigenomics	Biomarker discovery	MATLAB code https://sites.google.com/site/jointgenomics/
Rohart et al. (53)	44	2017	285	38.04	Feature transformation	Transcriptomics, genomics, proteomics, epigenomics	Subgroup identification	R package *Mixomics*
Sharifi-Noghabi et al. (54)	45	2019	2	6.91	Deep network	Genomics, transcriptomics	Subgroup identification (drug response)	https://github.com/hosseinshn/MOLI
Sehgal et al. (55)	46	2015	6	0.36	Network	Transcriptomics	Pathways analysis	
Song et al. (56)	47	2019	2	1.06	Feature transformation	Transcriptomics, genomics, proteomics	Biomarker discovery	R package *iProFun*
Speicher and Pfeifer (57)	48	2015	34	5.83	Clustering	Genomics, transcriptomics	Subgroup identification	
Vitali et al. (58)	49	2016	16	1.51	Network	Proteomics, transcriptomics	Drug repurposing/discovery	
Woo et al. (59)	50	2017	30	2.97	Clustering	Genomics, epigenomics	Subgroup identification	
Wu et al. (60)	51	2015	19	0.83	Clustering	Genomics, transcriptomics	Subgroup identification	
Yang et al. (61)	52	2019	2	1.23	Network	Epigenomics, transcriptomics	Biomarker discovery	
Yuan et al. (62)	53	2018	3	2.04	Network	Genomics, transcriptomics, epigenomics	Biomarker discovery	
Wang et al. (63)	54	2018	6	1	Network	Genomics, transcriptomics	Biomarker discovery	
Zhang et al. (64)	55	2018	9	1.58	Deep network	Transcriptomics, genomics	Subgroup identification	
Zhou et al. (65)	56	2015	2	0.18	Network	Genomics, epigenomics, proteomics	Biomarker discovery	
Zhu et al. (66)	57	2017	20	1.52	Feature transformation	Transcriptomics, genomics	Subgroup identification	
Žitnik and Zupan (67)	58	2015	14	2.50	Network	Transcriptomics, genomics	Biomarker discovery	

In the following sections, we describe the methodological categories that emerged from our literature review. For each methodological category, particular emphasis is placed on studies providing tools that can be exploited by other users, either with their own data or to reproduce their results.

### Network-Based Methods

Network-based approaches were exploited to detect, reconstruct and study interactions among sub network modules (13, 19, 22, 25, 40); to assess functional correlation among multi-omics entities (12, 14, 20, 55, 61, 62); to integrate and fuse networks to create comprehensive view of a disease (16, 24, 32, 37, 41, 63, 65). A few work leverage Bayesian methods (4, 34) or Markov models (17, 67).

Some approaches integrate network analysis within frameworks that apply multiple algorithms (35, 51, 58). In (51) a multi-platform analysis exploited for profiling pancreatic adenocarcinoma, includes clustering and Similarity Network Fusion to integrate genomic, transcriptomic, and proteomic data from the different platforms. In (58) authors develop a framework for drug repurposing and multi-target therapies by constructing a protein network for the disease under study and fusing several data sources. In (27), a functional interaction network predicts variations in expressions caused by genomic alterations, and it is exploited to prioritize cancer genes. Few others interesting approaches (16, 19) have been discussed in (10).

#### iOmicsPass

iOmicsPASS (40) implements a network-based method for integrating multi-omics profiles over genome-scale biological networks. The tool provides analysis components to transform qualitative multi-omics data into scores for biological interaction, then it uses the resulting scores as input to select predictive sub-networks; finally, it selects predictive edges for phenotypic groups using a modified nearest shrunken centroid algorithm. Authors validate iOmicsPASS on Breast Invasive Ductal Carcinoma data, integrating mRNA expression, and protein abundance, with and without the normalization of the mRNA data by the DNA Copy Number Variation (CNV). When compared with the original nearest shrunken centroid classification algorithm, iOmicsPASS outperform the baseline method, indicating the importance of selecting predictive signature forms densely connected sub networks, thus limiting the search space of predictive features to known interactions.

#### AMARETTO

Amaretto (22) is an algorithm developed multiple omics profiles integration across different type of cancers. Authors illustrate how the algorithm identifies cancer driver genes based on multi-omics data fusion and detects subnetworks of modules across all cancers. The algorithm identifies potential cancer driver genes by investigating significant correlations between methylation, CNV and gene expression (GE) data. When the driver genes are identified it constructs a module network connecting them with the co-expressed target genes they control. This constricts a pan-cancer network that is able to identify novel pancancer driver genes.

#### DrugComboExplorer

DrugComboExplorer (35) identifies candidate drug combinations targeting cancer driver signaling networks by processing DNA sequencing, CNV, DNA methylation, and RNA-seq data from individual cancer patients using an integrated pipeline of algorithms. The pipeline is based on two components: the first one extracts dysregulated networks from transcriptome and methylation profiles of specific patients using bootstrapping-based simulated annealing and weighted co-expression network analysis. The second component generates a driver network signatures for each drug, evaluates synergistic effects of drug combinations on different driver signaling networks and ranks drug combinations according the synergistic effects. In (35) authors apply DrugComboExplorer on diffuse large B-cell-lymphoma and prostate cancer, demonstrating the ability of the tool to discover synergistic drug combinations and its higher prediction accuracy compared with existing computational approaches.

### Deep Network

Deep Networks (DNs) are widely used to analyse omics-data (68). In a multi-omics scenario, clustering on DNs features showed different survival groups in neuroblastoma and liver cancer (23, 29, 64). In (42) authors integrated GE, methylation and miRNA in a restricted Boltzmann machine, where hidden layers represent different survival groups in breast cancer patients. Subnetworks are used in (54) to project different omics views in latent spaces that are further concatenated and fed into a final network to predict drug response.

#### SALMON

SALMON (Survival Analysis Learning with Multi-Omics Neural Networks) is a Deep Learning framework that integrates omics-data (mRNA and miRNA), clinical features and cancer biomarkers (36). Instead of feeding a neural network with mRNA and miRNA data, SALMON takes as input the eigengene matrices derived from co-expression analysis. Thus, it overcomes the high-dimensionality problem, reducing input features of about 99%. Authors assume that mRNA and miRNA data affect survival outcome independently, therefore the two corresponding eigengene matrices are connected to two different hidden layers whose output is linked to the final network with a Cox proportional hazards regression network. Results on breast cancer carcinoma patients showed improvements in survival prediction ability compared to single-omics.

### Clustering

Multi-omics clustering approaches are exploited to detect regularities and patterns that reveal different cancer molecular subtypes (21, 33, 43, 48, 57, 60) and prognostic groups in hepatocellular carcinoma (59). In (18) consensus clustering is performed on transcriptomics, metabolomics, and proteomics data to stratify patients with hepatocellular carcinoma based on their redox response. Clustering applications are often preceded by feature selection and/or feature transformation of multi-omics data, such as factorization, low rank approximation, and neural network. An exhaustive review on multi-omics integrative clustering approaches can be found in (69).

#### Nemo

NEMO (NEighborhood based Multi-Omics clustering) is a similarity-based tool that computes inter-patient similarity matrices for each omics through a radial basis function kernel. Spectral clustering is performed on the resulting average similarity matrix (52). NEMO addresses the problem of partial datasets, where not all the omics are measured for all the patients, and the final average matrix is computed on the observed omics values, without performing imputation. NEMO clustering shows higher performance compared to the same approach with imputed data, while on TCGA cancer datasets it detects significant differences in survival for six out of 10 cancer types.

#### Clusternomics

The main assumption of multi-omics clustering approaches relies on the existence of a consistent clustering structure across heterogeneous datasets. Alternatively, in (30) authors introduced the context-dependent clustering Clusternomics. Each omics is seen as a context describing a particular aspect of the underlying biological process. The global clustering structure is inferred from the combination of Bayesian clustering assignments. Then, by separating cluster assignment on two levels, Clusternomics allows the number of clusters to vary on local or global structure. Its performances are evaluated on a simulated dataset, where it showed higher Adjuster Rank Index compared to other clustering techniques, but also on breast, lung and kidney cancer from TCGA repository, where it identified clinically meaningful clusters.

#### Affinity Network Fusion

Affinity Network Fusion (AFN) (44) is both a clustering and classification technique that applies graph clustering to a patient affinity matrix incorporating information from multiple views. For each omic, after feature selection and/or transformation, AFN computes patient pair-wise distances. kNN Graph Kernel applied to the distance metric creates a patient affinity matrix for each view. The final affinity matrix is the weighted sum of the computed affinity matrices. AFN approach showed improved clustering performance in detecting cancer subtypes on several TCGA datasets when compared to its application in single omics.

### Feature Extraction

In multi-omics integration, variable selection to reduce the dimensionality of the omics dataset has a dominant role [(70), Figure 1A]. Recursive feature elimination was exploited to select subsets of expressed genes and methylation data to classify breast cancer disease subtypes with a Random Forest (3). Genes prioritization allowed prognosis prediction in different cancer types from epigenomics, transcriptomics, and genomics data (38), and biomarker discovery in prostate cancer (28). In (39) authors weight gene-gene interaction from transcriptomics and genomics data with a random walked-based method to select the most important interaction for survival prediction in breast cancer and neuroblastoma patients.

#### netDX

netDx is an algorithm that performs feature selection on Patient Similarity Networks (PSN) to classify patients in different prognostic groups (50). A PSN is built for each omics such that nodes represent patients and edges stand for the similarity of two nodes in the given view. Then netDx identifies which networks (i.e., which omics) strongly relate high- and low- risk patients through the GeneMANIA algorithm (71), which solves a regression problem to maximize the edges that connect query patients. Finally, each network is weighted according to its ability to relate patients of the same group and networks whose score exceeds a defined threshold are selected and combined in a single network by averaging their similarity scores. Authors benchmarked netDx against several machine-learning methods to predict survival outcomes on PanCancer TCGA multi-omics datasets, showing comparable results. On a breast cancer dataset, netDx selected features correspond to pathways known to be dysregulated in this type of cancer.

### Feature Transformation

Feature transformation (FT) refers to algorithms that replace existing features with new features still function of the original ones. As shown in Figure 1B, the majority of FT techniques aims at identifying cancer subtypes, biomarkers, omics-signatures, and key features from multi-omics data. Zhu et al. (66) proposed a kernel machine-learning method for a pan-cancer prognostic assessment by integrating multi-omics data. This work is particularly interesting since it's the only FT method we reviewed that allows multi-omics profile integration individually and in combination with clinical factors. A Kernel-based approach, combined with non-linear regression and Bayesian inference, resulted to be the best performing algorithm in a drug sensitivity prediction challenge (26).

In the following, we will report selected FT approaches, although few other tools for subgroup discovery, such as iClusterBayes (47), Multi-Omics Factor Analysis (15), JIVE (49), and MCIA (46), are available.

#### MixOmics

One of the most recent and biggest efforts in this field resulted in an R package called mixOmics (53). MixOmics allows for multivariate analysis of omics data including data exploration, dimension reduction, and visualization. mixOmics can be applied in numerous of studies with different aims such as integration and biomarker identification from multi-omics studies. The package includes two different types of multi-omics integration. One aimed at integrating different type of omics data of the same biological samples, while the second focus on integrating independent data measured on the same predictors to increase sample size and statistical power (53). Both frameworks aim at extracting biologically relevant features, [i.e., molecular signatures, by applying FT techniques (53)]. In (53) authors presented the results on 150 samples of mRNA, miRNA and proteomics breast cancer data and showed its ability to correctly discriminate three types of breast cancers.

#### mixKernel

mixKernel (45) is a R package compatible with mixOmics, which allows integration of multiple datasets by representing each dataset through a kernel that provides pairwise information between samples. The single kernels are then combined into one meta-kernel in an unsupervised framework. These new meta-kernels can be used for exploratory analyses, such as clustering or more sophisticated analysis to get insights into the data integrated. The authors showed better performances of mixKernel applied to mRNA, miRNAs and methylation breast cancer data if compared with one kernel approach.

#### iProFun

iProFun (56) is a method aimed at elucidating proteogenomic functional consequences of CNV and methylation alterations. The authors integrated mRNA expression levels, global protein abundances, and phosphoprotein abundances of a certain cancer. The output consists in a list of genes whose CNVs and/or DNA methylations significantly influencing some or all of the data integrated. iProFun obtains summary statistics of data integrated based on a gene-level multiple linear regression. These statistics are then used to extract genes having a cascading effect of all cis-molecular traits of interests and genes whose functional regulations are unique at global protein levels. iProFun applied to ovarian cancer TCGA dataset showed its ability in extracting interesting genes that could be considered targets for future therapies.

### Factorization

Traditional data mining methods are often inadequate to treat heterogeneous, sparse and noisy data such as multi-omics. Heavy pre-processing operations could modify, therefore loose, the inner structure of data coming from different sources. To discover latent characteristics hidden in huge amount of information, factorization techniques have been applied to highlight complex interactions among omics-data, hard to detect using standard approaches.

Gao et al. (31) developed an integrated Graph Regularized Non-negative Matrix Factorization model focused network construction by integrating gene expression data, CNV data, and methylation data. The authors used the factorization technique to decompose and fuse the multi-omics data. Then, by combining the results with network and mining analyses they showed how their method was able to find potential new cancer-related genes on two different TCGA datasets. Another method, based on factor analysis, aims at identifying latent factors in the multi-omics-data integrated in the model that can be used for subsequent analysis such as subgroup identification (15). Give its aim in extracting hidden features, we described this method in detail in the feature transformation section.

## Discussion

Along with technological advances in high-throughput sequencing, which characterize multiple “omes” from biological samples, holistic systems for data integration and knowledge discovery with machine-learning algorithms are still under development. Precision oncology would greatly benefit from actionable knowledge gained from multi-omics assays. In this paper we provided an overview of recent works on this topic and highlight current achievements and limitations.

We reviewed relevant tools to perform analysis based on different combination of omics, and observed their growing numbers in recent years, indicating strong commitments to develop such tools. Several issues emerged, too. The majority of the proposed techniques were applied to TCGA dataset, and data integration was mainly focused on transcriptomics and genomics. Efforts should be devoted to make new data sources available to the research community (72), such as the UKBioBank (73) and DriverDBv3 (74), and to integrate other “omes,” such as metabolome, or patient-generated, and environmental data. Research in this field would greatly benefit from the development of databases specifically developed for containing and facilitating the analysis of multi-omics and clinical data, such as LinkedOmics (75). Another important improvement to increase usability and reproducibility would be to aim at developing methods that can be applied and generalized for all omics data type.

The complexity of multi-omics data analysis requires collaborative efforts among the clinical and machine-learning communities and the joint application of methodologies derived from heterogenous backgrounds. We noted that some promising methods, such as matrix-factorization have not been extensively exploited, while clustering and network-based approaches are the most extensively used, probably due to their flexibility and the possibility to be integrated in comprehensive frameworks that include feature extraction and transformation to deal with the curse of dimensionality. Deep learning methods, that are flexible and achieved outstanding results in other fields, are increasingly used, even though many works share the same “pipeline” (i.e., the exploitation of autoencoder hidden layers for clustering). Interestingly, the number open source tools have increased in the very last years (Figure 1B).

We are aware of some limitations of our review. An important aspect that has not been covered by this review is the quantitative comparison among tools (76), which could highlight possible overfitting (77) and issues that may prevent the actual translation of multi-omics approaches from bench to bedside. Although, by indicating works that provide a usable tool (Table 1), our review could be a starting point for a comprehensive quantitative comparison.

## Author Contributions

RB conceived the study. GN, FV, and AD run the analyses and wrote the article. NG and RB revised the article. All authors contributed to the article and approved the submitted version.

## Conflict of Interest

The authors declare that the research was conducted in the absence of any commercial or financial relationships that could be construed as a potential conflict of interest.
